# Supplementary material for: Gallium-Doped Hydroxyapatite Shows Antibacterial Activity against Pseudomonas aeruginosa without Affecting Cell Metabolic Activity
Source: J Funct Biomater. 2023 Jan 17;14(2):51. doi: 10.3390/jfb14020051 (PMC9961062; doi:10.3390/jfb14020051)
Supplement: Supplementary file 1 [file jfb-14-00051-s001.zip › jfb-2131344-supplementary.pdf]

Supplementary data

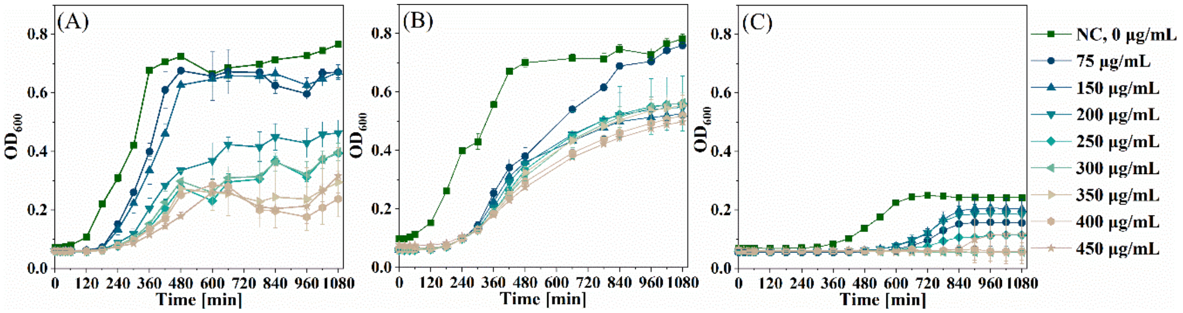

Figure S1. Growth curves in the presence of Ga(NO3)3*4.2H2O at different concentrations of (A) *E. coli* (B) *S. epidermidis* and (C) *S. pyogenes*

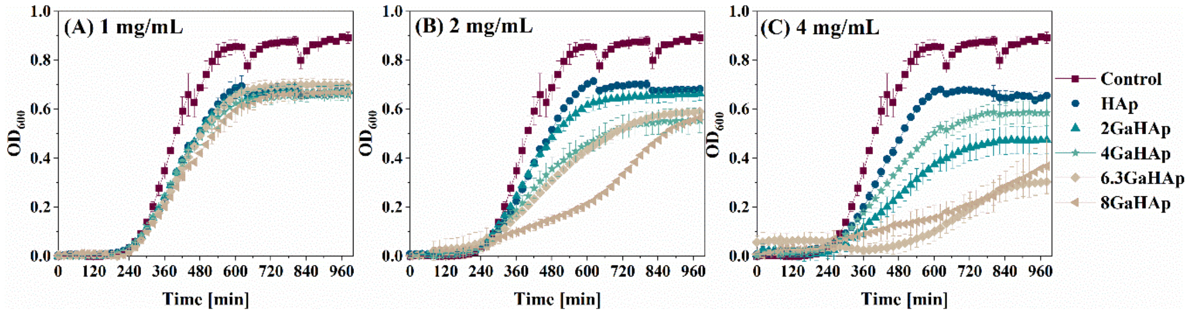

Figure S2. *S. epidermidis* growth curves in the presence of GaHAp powder suspension in TSB at different concentrations

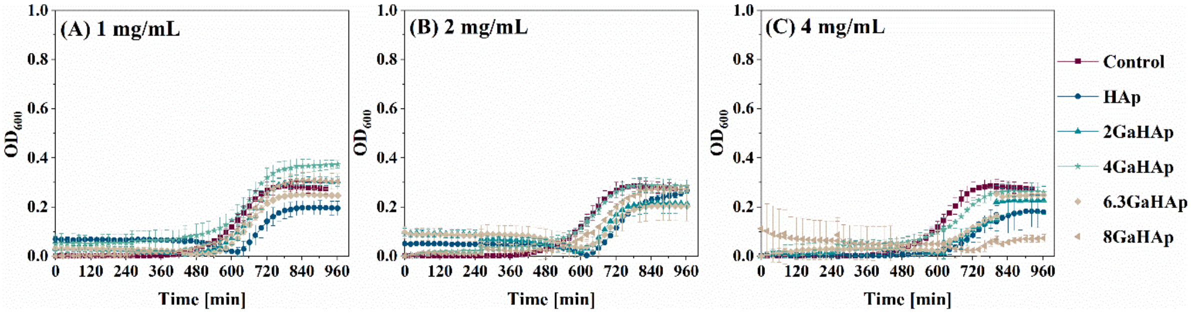

Figure S3. *S. pyogenes* growth curves in the presence of GaHAp powder suspension in TBS at different concentrations

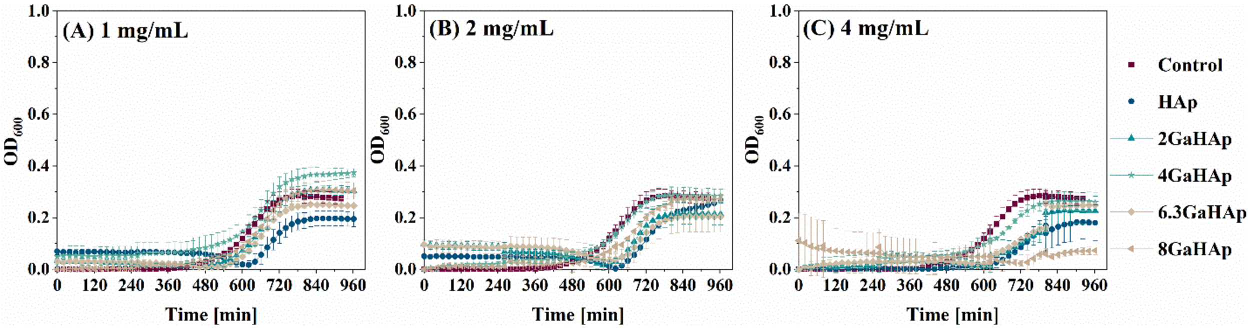

Figure S4. *E. coli* growth curves in the presence of GaHAp powder suspension at different concentrations

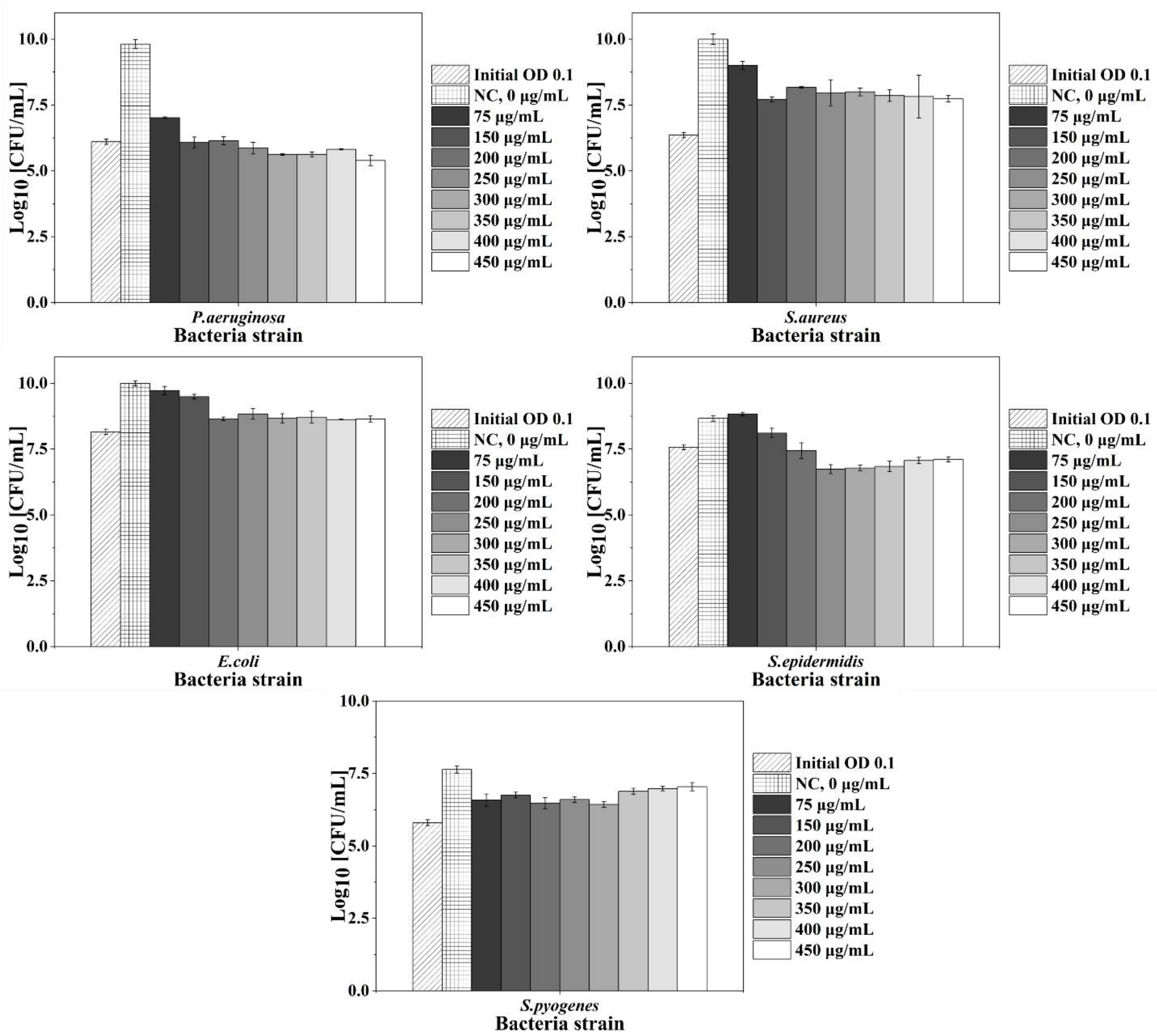

Figure S5. The minimal inhibitory concentration of $Ga(NO_3)_3$*$4.2H_2O$ against 5 bacteria species

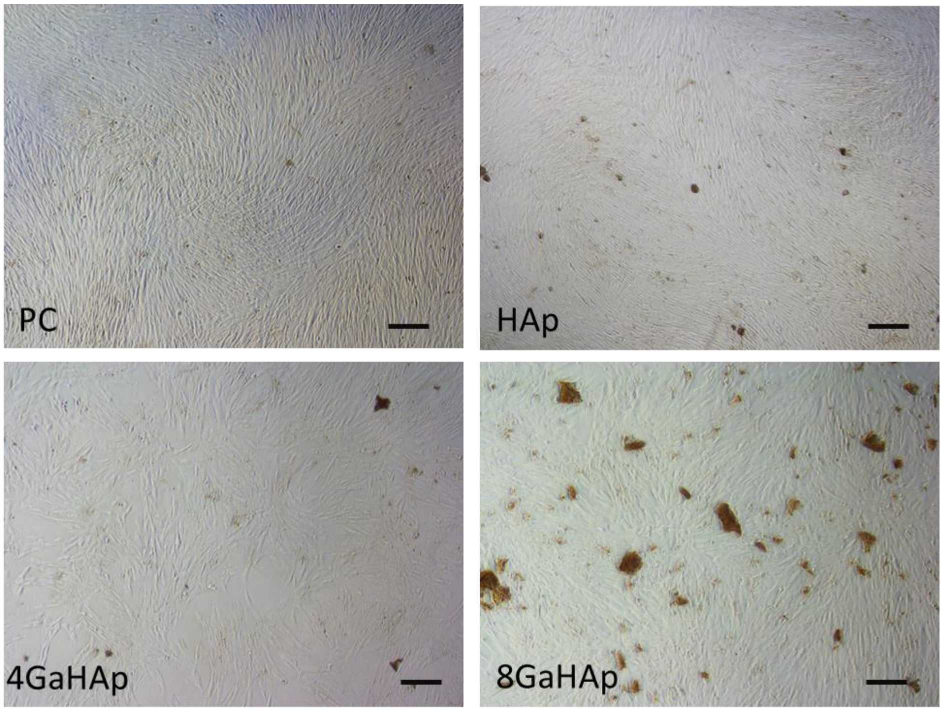

Figure S6. Human fibroblasts (hTERT-BJ1) were exposed to GaHAp by indirect test on day 3. PC-positive control; scale bar – 200 µm.

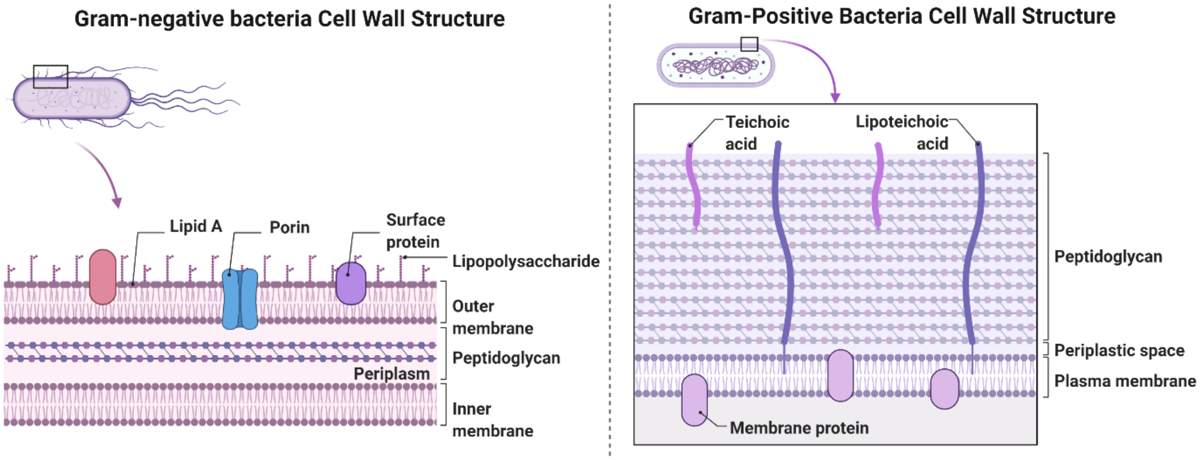

Figure S7. The structural difference between Gram-negative and Gram-positive bacteria (created by Biorender.com)
